# Effects of Sildenafil on the Gastrocnemius and Cardiac Muscles of Rats in a Model of Prolonged Moderate Exercise Training

**DOI:** 10.1371/journal.pone.0069954

**Published:** 2013-07-29

**Authors:** Barbara Rinaldi, Maria Donniacuo, Loredana Sodano, Giulia Gritti, Simona Signoriello, Elisabetta Parretta, Liberato Berrino, Konrad Urbanek, Annalisa Capuano, Francesco Rossi

**Affiliations:** 1 Department of Experimental Medicine, Section of Pharmacology “L. Donatelli”, Centre of Excellence for Cardiovascular Diseases, Second University of Naples, Naples, Italy; 2 Department of Experimental Medicine, Section of Pharmacology “L. Donatelli”, Regional Centre for Pharmacovigilance and Pharmacoepidemiology, Second University of Naples, Naples, Italy; 3 Department of Mental and Physical Health and Preventive Medicine, Medical Statistics, Second University of Naples, Naples, Italy; University Heart Center Freiburg, Germany

## Abstract

Moderate exercise training improves energetic metabolism, tissue perfusion and induces cardiac and skeletal muscle remodeling. Sildenafil, a potent phosphodiesterase-5 inhibitor used to treat erectile dysfunction, reduces infarct size and increases tissue oxygenation in experimental models of cardiovascular disease. We have evaluated the effects of prolonged moderate exercise training and a repeat administration of sildenafil on the rat gastrocnemius and cardiac muscles. Animals were divided into two groups: sedentary and trained. Each group was subdivided into animals treated with vehicle or with two doses of sildenafil (10 or 15 mg/kg/day) during the last week of training. Physical exercise did not induce cardiac hypertrophy, whereas it increased mRNA levels of the PGC-1α, HIF-1α and VEGF genes, which are involved in mitochondrial biogenesis and angiogenesis, and reduced mRNA levels of FoxO3a, MuRF-1 and Atrogin-1. Sildenafil dose-dependently promoted both angiogenesis, as shown by increased capillary density, and muscle atrophy, as shown by muscle fibre size. These effects were more pronounced in trained animals. Our data confirm the beneficial effects of a moderate and prolonged training on cardiovascular and skeletal systems and document the positive and negative effects of sildenafil on these tissues at doses higher than those used in clinical practice. This report may impact on the use of sildenafil as a substance able to influence sports performance.

## Introduction

It is widely recognized that exercise training can improve cardiac and skeletal performance [1], [2]. In fact, a large body of evidence suggests that regular physical activity induces marked vascular remodeling in skeletal muscle and myocardium by increasing angiogenesis and arteriogenesis, myocardial perfusion, energetic metabolism, oxygen uptake, substrate oxidation and resistance to fatigue [3]–[5]. These effects are mediated by the activation of several signalling pathways involved in the maintenance of energy homeostasis and mitochondrial biogenesis [6]. In particular, clinical and experimental studies have demonstrated that exercise training increases nitric oxide (NO) bioavailability by readjusting the balance between NO generation/inactivation and reducing oxidative stress [7], [8].

Sildenafil is a potent inhibitor of phosphodiesterase-5 (PDE5) that has been used in the management of erectile dysfunction [9].

PDE5 is expressed in vascular and bronchial smooth muscle, platelets, cardiomyocytes and skeletal muscle [10], [11]. By increasing cGMP concentration and modifying NO availability and/or biological activity, PDE5 causes vasodilatation [12], [13]. Moreover, in a recent double-blind, cross-over trial, Di Luigi *et al*. [14] demonstrated that the administration of another PDE5 inhibitor, tadalafil, reduced activation of the hypothalamus-pituitary-adrenal axis during exercise probably by influencing brain NO and its pathway.

Powerful basic research and clinical studies suggest new potential applications of PDE5 inhibitors mainly in cardiovascular diseases, namely, ischemia and heart failure [15]–[17]. Moreover, sildenafil has been shown to ameliorate exercise capacity both in subjects affected by cardiopulmonary diseases in normoxia [18], and in healthy subjects in hypoxia [19]. Interestingly, although sildenafil does not have an indication for use in equine veterinary medicine, in 2000 it was the eighth most frequent drug found in urine samples from racing horses, which suggests its use in doping [20].

The peroxisome proliferator-activated receptor γ co-activator-1 alpha (PGC-1α) belongs to a family of transcription factors induced by an hypoxic condition that regulate mitochondrial biogenesis through the expression of genes encoding proteins involved in the maintenance of glucose, lipid and energy homeostasis in muscle and in other mitochondrial-rich tissues [5], [21], [22]. Moreover, PGC-1α is strongly induced by both short-term and chronic exercise in rodents and humans. This suggests that PGC-1α is involved in several pathways activated during exercise and, consequently, that it plays a functional role in exercise-induced muscle adaptation [23], [24]. In fact, PGC-1α is an important regulator of transcription of vascular endothelial growth factor (VEGF) and forkhead box (Fox)-O, which are involved in angiogenetic and atrophic processes, respectively [25], [26]. In particular, the nuclear accumulation of the FoxO3a factor and the expression of Atrogin-1 and muscle-specific ring finger protein (MuRF-1) lead to an increase in protein degradation and muscle atrophy [27]. In this context, it is noteworthy that transgenic overexpression of PGC-1α protects skeletal muscle from atrophy by suppressing FoxO3a activity [28], [29]. Moreover, it is well known that intracellular hypoxia inducible factor-1 alpha (HIF-1α) is activated during exercise and that this is one of signalling that induces activation of VEGF expression and hence angiogenesis [25], [30].

In this scenario, we evaluated the effects of prolonged moderate exercise training, and the effects of a repeat administration of sildenafil on the gastrocnemius and cardiac muscles of rats. Specifically, we evaluated atrophy and angiogenesis phenomena to determine whether sildenafil induces skeletal and cardiac modifications.

## Materials and Methods

### Animals

Male Wistar rats (200–250 g) purchased from Harlan Italy (San Pietro al Natisone, Udine, Italy) were housed in individual cages under controlled conditions (12–12 h light-dark cycle; room temperature 20±22°C; humidity 55–60%) with chow and tap water available *ad libitum.* All experimental procedures were approved by the Animal Ethics Committee of the Second University of Naples. Animal care was in compliance with Italian (Decree 116/92) and European Community (E.C. L358/1 18/12/86) guidelines on the use and protection of laboratory animals. All efforts were made to minimize animal suffering and to reduce the number of animals used.

**Figure 1:**
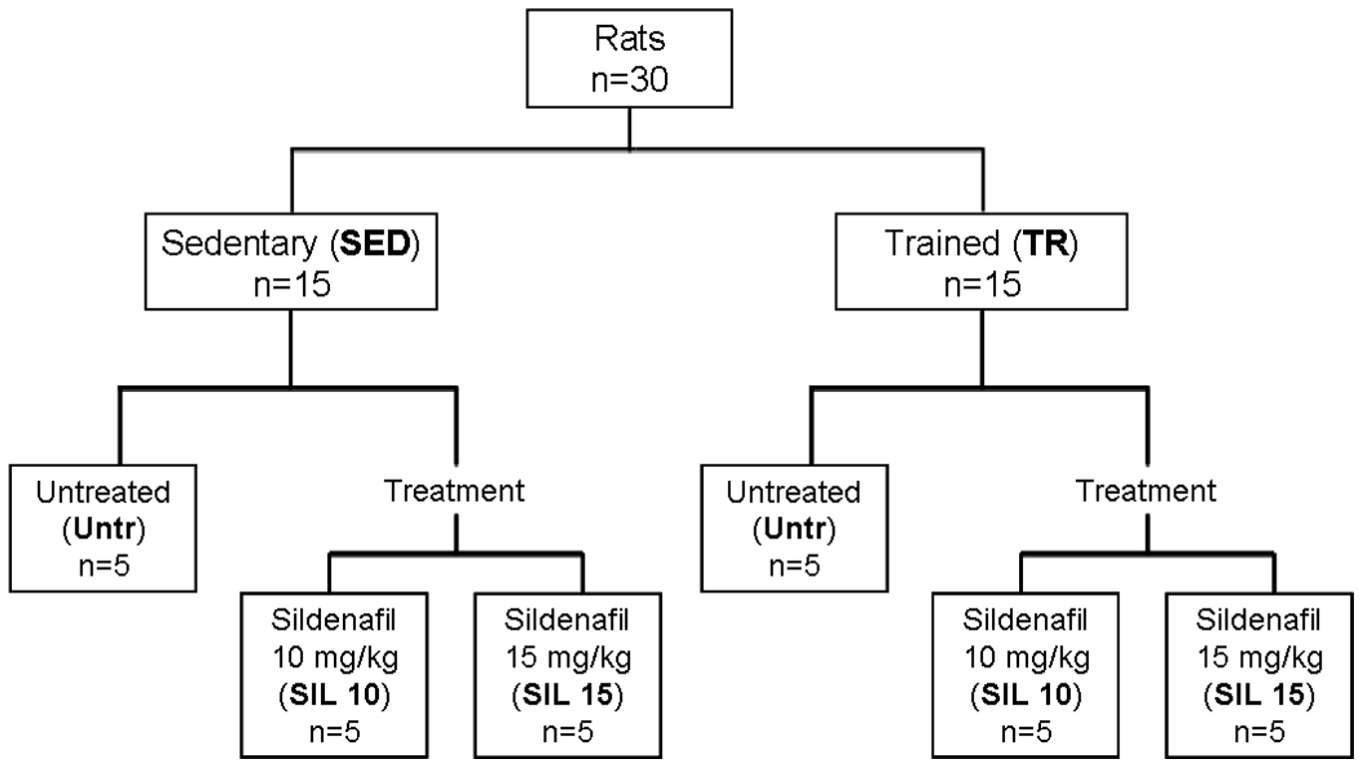
Flow diagram showing the experimental groups.

Thirty rats were allocated to two main groups: sedentary (SED; n = 15) and exercise trained (TR; n = 15). Rats were randomly divided into three subgroups: an untreated group (Untr) consisting of sedentary (n = 5) or trained (n = 5) rats that received vehicle (saline solution 0.9% NaCl dissolved in 5% dimethyl sulfoxide [DMSO; Sigma-Aldrich Company, Milan, Italy]), and two treated groups consisting of sedentary (n = 10) or trained (n = 10) rats treated with 10 or 15 mg/kg/day sildenafil citrate (SIL 10 and SIL 15, respectively; Figure 1). Vehicle or sildenafil (Pfizer Inc., Groton, CT, USA) was administered by subcutaneous injection during the last week of the training protocol. As for other performance enhancing drugs, sildenafil was used at doses higher than those used in clinical practice.

### Training Protocol

The training protocol was performed as previously described [31]. Briefly, the three groups of trained rats were acclimated to training by walking at a speed of 10 min/day on a treadmill for 2 weeks (Panlab/Harvard Apparatus Treadmills, Holliston, MA, USA). From week 3, training consisted of running of 30 m/min, 45 min/day, 5 days/week, for 6 weeks. A horizontal shock grid that delivered <1 mA was placed 10 cm from the rear of the chamber to provide stimulus for the animals to run. In fact, rats were placed on the grid treadmill cell and exercise capacity of each rat was measured by the effective time of exercise calculated as the difference between the total running time of each rat and the time spent on the shock grid [32].

### Hemodynamic Measurements

Mean arterial blood pressure (MAP) and heart rate (HR) were measured before, during (week 3) and at the end of the study in all 30 animals using the non invasive tail-cuff system (model BP 2000 Blood Pressure Analysis System, Visitech Systems; Apex, NC, USA) [33]. Between 5 and 7 days before the experiment, rats were acclimated to restraint and tail-cuff inflation. The restraint platform was maintained at 33–34°C. For each MAP measurement, the rat was placed in a metal box restraint, and a cuff occluder with a pneumatic pulse sensor was attached to the tail. On inflation, the occluder stopped blood flow through the tail and on deflation the return of blood flow was detected by the sensor. An initial series of inflation-deflation cycles was used to set amplifier and instrument controls. This instrument automatically takes 10 30-s measurements using proprietary software (BP-2000 Software Beta Version 03/10/97). If at least 8 of 10 readings were acceptable, the highest and lowest readings were discarded, and the remaining readings were averaged for a single session value. Only the values of MAP and HR measured at the end of the study were reported in the Results section.

### Transthoracic Echocardiography Measurements

Left cardiac morphology and function were evaluated using a non invasive transthoracic M-Mode echocardiography (VisualSONICS VeVo 770 imaging system with a RMV710B; Toronto, Ontario, Canada) in rats anesthetized with an intraperitoneal injection of ketamine hydrochloride (100 mg/kg) and xylazine (2.5 mg/kg) supplemented as needed. Transthoracic echocardiographic determinations were performed in all 30 animals in the lateral decubitus position before, during (week 3 of the study), and at the end of the study. Only the determinations at the end of the study were reported in the Results section.

### Real-time Reverse Transcriptase PCR

Total RNA was isolated from the gastrocnemius and heart muscles of all rats. Frozen tissues were powdered and homogenized with l ml of TRIzol twice using a rotator-stator and a 23 G needle (Invitrogen, Milan, Italy) according to the protocol recommended by the manufacturer and described elsewhere [34]. Using cDNA obtained by reverse transcriptase of RNA extracted from frozen tissues (Bio-Rad Laboratories, Milan, Italy), the expression levels of FoxO3a, Atrogin-1, MuRF-1, PGC-1α, VEGF, HIF-1α and Glyceraldehyde 3- phosphate dehydrogenase (GAPDH) mRNAs were quantified by real-time RT-PCR using SYBR Green (Bio-Rad Laboratories, Milan, Italy). The oligonucleotide sequences used as primers are listed in Table 1. mRNA concentrations were expressed as ratio over GAPDH which was amplified as housekeeping gene.

**Table 1:** Primer sequences used for real-time PCR mRNA analyses.

Gene Name (symbol)	GenBank accession no.	PCR primer sequence 5′→ 3′	Amplicon length (bp)
GAPDH	NM_017008	Fwd: GCATCCTGCACCACCAACTG Rev: CACAGTCTTCTGAGTGGCAG	117
FoxO3a	NM_001106395	Fwd: GTCCCTGAAGGGAAGGAGC Rev: CTCGTCCAGGATGGCGTAG	111
Atrogin-1	NM_133521	Fwd: GGAGCTGATAGCAAAGTCAC Rev: GGAGAAGTTCCCGTATGAGTC	134
MuRF-1	NM_080903	Fwd: CTCGCAGCTGGAGGACTCC Rev: CTCGTCCAGGATGGCGTAG	103
PGC-1α	NM_031347	Fwd: CACCAAACCCACAGAGAACAG Rev: GGTGACTCTGGGGTCAGAG	135
VEGF	NM_031836	Fwd: CTTTCTGCTCTCTTGGGTGC Rev: GTAGACGTCCATGAACTTCAC	133
HIF-1α	NM_024359	Fwd: CCCATTCCTCATCCATCAAAC Rev: TCTTCTGGCTCATAACCCATC	131

### Western Blot Analysis

Gastrocnemius and cardiac muscle tissues were analyzed to investigate FoxO3a, Atrogin-1, MuRF-1 and PGC-1α protein expression. Gastrocnemius muscle was homogenized on ice with a buffer containing 50 mM Tris-HCl (pH 7.4), 150 mM NaCl, 5 mM EDTA, 0.5% SDS, 1% sodium deoxycolate, 0.1% Triton X-100, 0.05% β-mercaptoethanol. Heart samples were homogenized on ice using RIPA lysis buffer (Santa Cruz Biotechnology, Milan, Italy). In each buffer was added a cocktail of protease and phosphatase inhibitors. The lysates were collected and centrifuged at 13,000 g for 10 min to remove the insoluble debris. Protein concentration of the resulting supernatant was determined using a commercial kit (Bio-Rad Laboratories, Milan, Italy). Equal amounts of proteins were separated on denaturing 10% SDS polyacrylamide gel and transferred to a nitrocellulose membrane. Membranes were blocked for 1 h at room temperature with 5% milk in T-TBS (Tris buffer saline with 0.1% Tween 20), followed by incubation at 4°C overnight with primary antibodies against FoxO3a (#47409, Abcam, Cambridge, UK), Atrogin-1, MuRF-1 and PGC-1α (#33782, #27642, #13067 respectively; Santa Cruz Biotechnology, Milan, Italy). Membranes were then washed three times with 0.1% T-TBS solution, and incubated for 1 h at room temperature with a secondary antibody goat anti-rabbit IgG-HRP and donkey anti-goat IgG-HRP (#2004 and #2020 respectively; Santa Cruz Biotechnology, Milan, Italy).

GAPDH antibody (#25778; Santa Cruz Biotechnology, Milan, Italy) was used as an internal standard. The immunoreactive bands were visualized using an enhanced chemiluminescence system (SuperSignal West Femto Maximum Sensitivity Substrate, Pierce, Rockford, USA). The protein bands were scanned and quantitated with ChemiDoc-It® 500 Imaging System UVP (Upland, CA, USA).

### Immunohistochemistry

Samples of gastrocnemius and cardiac muscle tissue were fixed by immersion in 10% phosphate-buffered formalin (Sigma-Aldrich Company, Milan, Italy). Tissue specimens were embedded in paraffin and 5 µm thick histological sections were obtained for immunohistochemistry. Sections were deparaffinized and, after rehydration and incubation with 10% normal donkey serum, they were incubated overnight with primary antibodies. Capillaries were identified by the expression of platelet endothelial cell adhesion molecule (PECAM)-1 using the goat polyclonal antibody (Santa Cruz Biotechnology, Milan, Italy); myocytes were labelled with a mouse anti-α-sarcomeric actin antibody (Sigma-Aldrich Company, Milan, Italy). The expression of FoxO3a was determined by labelling with a rabbit polyclonal antibody (Abcam, Cambridge, UK). Secondary antibodies conjugated with fluorescein isothiocyanate (FITC), tetramethyl rhodamine isothiocyanate (TRITC) and Alexa 594 (Jackson ImmunoReaserch, Milan, Italy) were used to detect primary antibodies. The number of FoxO3a-labelled myocyte nuclei was manually counted in a blinded fashion with a Leica DM5000B fluorescence microscope at 1000x magnification in randomly selected fields and the percentage of positive cells was calculated. The number of capillary profiles was counted with a Zeiss LSM510 Meta confocal microscope (Carl Zeiss, Milan, Italy) using a 63x oil-immersion objective in 10 microscope fields per each sample. After immunostaining with an anti-α-sarcomeric actin antibody, muscle fibres were measured with Image Pro Plus software (Media Cybernetics, Bethesda, MD, USA) on the images with the dimensions of 900×900 µm. To assure the accuracy of the measurements and to avoid the potential mistakes related to muscle fibres orientation, the images were recorded in the areas where not tilted cross-sectional area of a gastrocnemius muscle fibres was clearly visible.

### Statistical Analysis

Physical and haemodynamic data were reported as mean ± SD. Each variable of training and sildenafil effects were analysed by two-way ANOVA and tested for interaction. The overall sildenafil effect was assessed by one-way ANOVA followed by pairwise comparisons between doses with the Tukey’s test to adjust for multiple comparisons. If the interaction between training and sildenafil doses from two-way ANOVA was statistically significant (p<0.05), results of Tukey’s test were reported separately in the two groups (trained or sedentary), otherwise results are reported for the two groups combined.

Gastrocnemius and cardiac gene expressions data were analysed similarly; however since no reliable information on the gaussianity assumption previously available, a robust rank-based non parametric alternative was rather used [35]. Tables reported median values and inter-quartiles ranges for all groups, and in the figures the observed individual values were depicted.

A repeated measure ANOVA were performed to evaluate the observed exercise times in the three groups of trained rats.

## Results

### Effects of Sildenafil on Exercise Capacity

**Figure 2:**
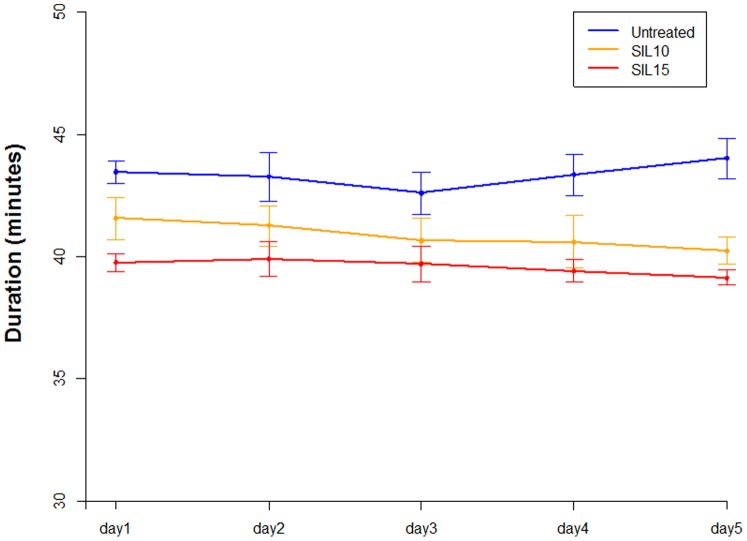
Effects of sildenafil treatment on rats exercise capacity. For each day, average duration of exercise (minutes) is reported for each experimental group. Vertical bars represent standard deviation (SD).

Effects of sildenafil on exercise capacity are reported in Figure 2. Time duration of running was significantly reduced by increasing doses of sildenafil (p<0.001) while no significant difference was found between days (p = 0.19).

### Effects of Sildenafil on Physical and Haemodynamic Parameters in Trained and Untrained Rats

Table 2 shows the physical and haemodynamic data obtained at the end of the 8-week training protocol; the data obtained at the earlier time points were not significantly different. As expected, trained rats were leaner than sedentary rats independently of treatment (interaction p-value = 0.54); sildenafil treatment had no effect on body weight (p = 0.14).

**Table 2:** Physical and hemodynamic data.

	Sedentary Untreated (n = 5)	Sedentary SIL10 (n = 5)	Sedentary SIL15 (n = 5)	Trained Untreated (n = 5)	Trained SIL10 (n = 5)	Trained SIL15 (n = 5)
**Physical data**						
Body wt (g)	443±8.5	448.3±10	445±3	363.33±8^b^	373±10^b^	375±13^b^
Heart wt (g)	0.93±0.13	1.03±0.08	0.92±0.04	0.95±0.05	0.95±0.06	1.01±0.07
aHeart wt/body wt (g/kg)	2.09±0.001	2.29±0.001^§^	2.06±0.001^§,°^	2.6±0.001^b^	2.54±0.0001^b,§^	2.69±0.001^b,§,°^
aTibial length (cm)	4±0.2	4.2±0.1*	3.8±0.15*^,°^	3.9±0.05	4±0.01	4±0.2
Heart wt/tibial length (g/cm)	0.25±0.02	0.23±0.04	0.24±0.03	0.24±0.02	0.24±0.03	0.25±0.04
**Haemodynamic data**						
Heart rate (bpm)	458±1.4	455±5.6	450.5±6.3	440.5±2.1^b^	438±2.9^b^	441±0.7^b^
MAP (mmHg)	135±1	124.4±2.08^§^	114.7±1.5^§,°^	137.7±2	123±1^§^	117±2.6^§,°^
EF (%)	80±0.8	82±0.3*	82.5±0.3*	83.6±1.7^b^	87.3±1^b,^*	88.1±0.6^b,^*

Abbreviations: MAP, mean arterial blood pressure; EF, ejection fraction. Data are mean ± SD.

a Interaction between training and sildenafil dose was statistically significant (sildenafil dose effect must be read separately for sedentary and trained rats).

Training effect: ^b^p<0.001 Trained vs. Sedentary.

Sildenafil dose effect: ^§^p<0.001 vs. Untreated, *p<0.05 vs. Untreated, ^°^p<0.001 vs. SIL10.

As shown in Table 2, heart weight/tibia length, as a measure of cardiac hypertrophy, was similar in trained and untrained rats. Moreover, exercise training did not affect MAP (p = 0.11) that was significantly reduced by sildenafil (p<0.001, for all comparisons). On the other hand, as expected, training significantly reduced HR (p<0.001) but there was not a dose effect (p = 0.16). Finally, both training and treatment with sildenafil significantly increased ejection fraction (EF) but no difference was found in the comparison of SIL 15 vs. SIL 10 (p = 0.56).

### Effects of Sildenafil on Gastrocnemius and Cardiac Muscle in a Rat Model of Prolonged Moderate Exercise

**Figure 3:**
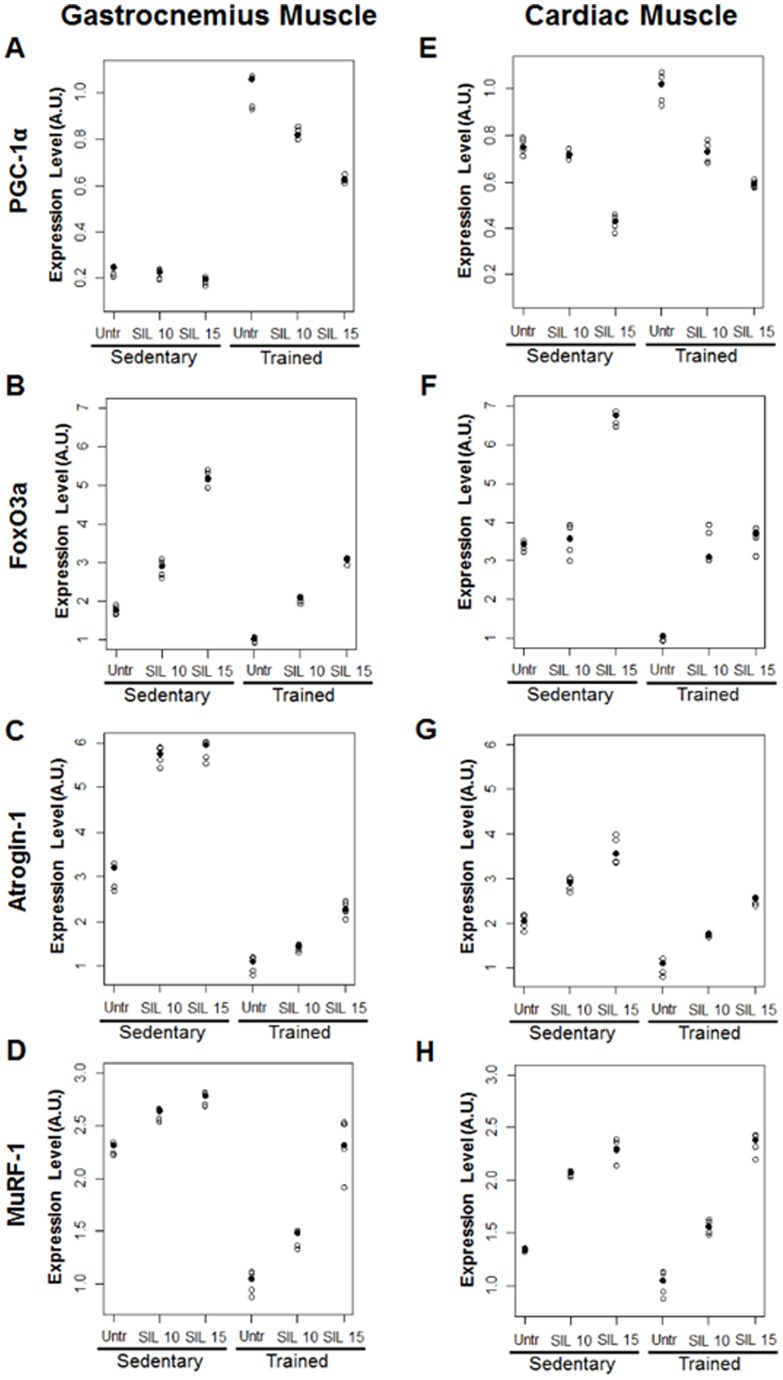
Effects of sildenafil on skeletal and cardiac muscle atrophy. PGC-1α, FoxO3a, Atrogin-1 and MuRF-1 gene expression was measured by real-time PCR. Left panel (A–D) gastrocnemius gene expression; right panel (E–H) cardiac gene expression. Individual observed values are plotted by study groups. Median values are highlighted by solid circles.

Results of training and sildenafil doses on gastrocnemius and cardiac muscle, measured by RT-PCR, are reported in Table 3 and Figure 3. Sildenafil doses were tested separately in the two groups in all cases but for Atrogin-1, in cardiac muscle, the interaction between training and sildenafil dose was not statistically significant.

**Table 3:** Effects of sildenafil on skeletal and cardiac muscle atrophy.

	Sedentary Untreated (n = 5)	Sedentary SIL10 (n = 5)	Sedentary SIL15 (n = 5)	Trained Untreated (n = 5)	Trained SIL10 (n = 5)	Trained SIL15 (n = 5)
**Gastrocnemius muscle**						
aPGC-1α	0.25 (0.22–0.25)	0.23 (0.20–0.24)	0.20 (0.18–0.20)*^†^	1.06 (0.94–1.06)^b^	0.82 (0.82–0.84)^b§^	0.63 (0.62–0.65)^b§°^
aFoxO3a	1.77 (1.71–1.85)	2.91 (2.71–3.03)^§^	5.19 (5.14–5.33)^§°^	1.02 (0.95 1.05)^b^	2.09 (2.00–2.10)^b§^	3.06 (2.94–3.10)^b§°^
aAtrogin-1	3.22 (2.77–3.29)	5.75 (5.61–5.87)^§^	5.95 (5.66–6.00)^§^	1.10 (0.90–1.18)^b^	1.42 (1.36–1.46)^b^*	2.28 (2.23–2.39)^b§°^
aMuRF-1	2.32 (2.24–2.34)	2.64 (2.56–2.65)^§^	2.78 (2.70–2.80)^§°^	1.05 (0.95–1.10)^b^	1.48 (1.36–1.51)^b§^	2.32 (2.28–2.51)^b§°^
**Cardiac muscle**						
aPGC-1α	0.75 (0.74–0.78)	0.72 (0.71–0.72)	0.43 (0.41–0.45)^§°^	1.02 (0.95–0.05)^b^	0.73 (0.69–0.76)^b§^	0.59 (0.58–0.60)^b§†^
aFoxO3a	3.45 (3.32–3.51)	3.59 (3.29–3.85)	6.76 (6.56–6.86)^§°^	1.05 (0.95–1.07)^b^	3.10 (3.09–3.71)^b§^	3.69 (3.60–3.74)^b§†^
Atrogin-1	2.05 (1.95–2.16)	2.91 (2.78–2.98)*	3.55 (3.38–3.86)^§†^	1.10 (0.90–1.10)^b^	1.73 (1.72–1.75)^b^*	2.55 (2.45–2.58)^b§†^
aMuRF-1	1.34 (1.34–1.35)	2.08 (2.04–2.09)^§^	2.30 (2.28–2.36)^§°^	1.05 (0.95–1.12)^b^	1.56 (1.51–1.60)^b§^	2.38 (2.32–2.42)^b§°^

Data are reported as median and interquartile range.

a Interaction between training and sildenafil dose was statistically significant (sildenafil dose effect must be read separately for sedentary and trained rats).

Training effect: ^b^p<0.001 Trained vs. Sedentary.

Sildenafil dose effect: ^§^p<0.001 vs. Untreated, *p<0.05 vs. Untreated, ^°^p<0.001 vs. SIL10, ^†^p<0.05 vs. SIL10.

Sildenafil treatment counteracted in a dose-dependent manner the beneficial effects of training on atrophy in both gastrocnemius and cardiac muscle reducing the expression of PGC-1α, an atrophy protective gene, and increasing the expression of the “atrogenes” FoxO3a, Atrogin-1 and MuRF-1. Eight weeks of exercise training induced PGC-1α gene expression, measured by RT-PCR, in the gastrocnemius (p<0.001) and cardiac muscles (p<0.001) (Figure 3A, E). Treatment with sildenafil (10 or 15 mg/kg/day) induced a significant, dose-dependent reduction of PGC-1α mRNA in both tissues of trained rats (gastrocnemius: p<0.001; heart: p<0.001), but in a different manner in trained and sedentary group (interaction test p<0.001). In sedentary rats this effect was evident only after administration of the highest dose of sildenafil (p<0.001) with a significant difference also between the two doses (p<0.001) (Figure 3A, E). As expected, exercise training reduced mRNA expression of FoxO3a (Figure 3B, F) and consequently Atrogin-1 (Figure 3C, G) and MuRF-1 gene expression (Figure 3D, H) in gastrocnemius and cardiac muscles (p<0.001). Treatment with sildenafil induced a dose-dependent increase of FoxO3a in both tissues (p<0.001) but in a different manner in trained and sedentary group (interaction test p<0.001). In cardiac muscle of sedentary rats there was no significant difference between lower dose of sildenafil and untreated (p = 0.60) (Figure 3F). In gastrocnemius muscle all the comparisons between doses in sedentary and trained groups were statistically significant (p<0.001) (Figure 3B). Treatment with sildenafil induced a dose-dependent increase of Atrogin-1 in cardiac tissue in the same way in trained and sedentary group (interaction test p = 0.52) and the comparison of all doses was statistically significant (Figure 3G). In gastrocnemius muscle sildenafil induced a dose-dependent increase of Atrogin-1 in a different manner in trained and sedentary group (interaction test p<0.001). In sedentary group no difference was found between lower and higher dose (p = 0.73) (Figure 3C). Similar results were found for MuRF-1 in both tissues (interaction test p<0.001) and all the comparisons between doses were statistically significant (p<0.001).

### Effects of Sildenafil on PGC-1α, FoxO3a, Atrogin-1 and MuRF-1 Expressions in Gastrocnemius and Cardiac Muscle after Exercise Training

**Figure 4:**
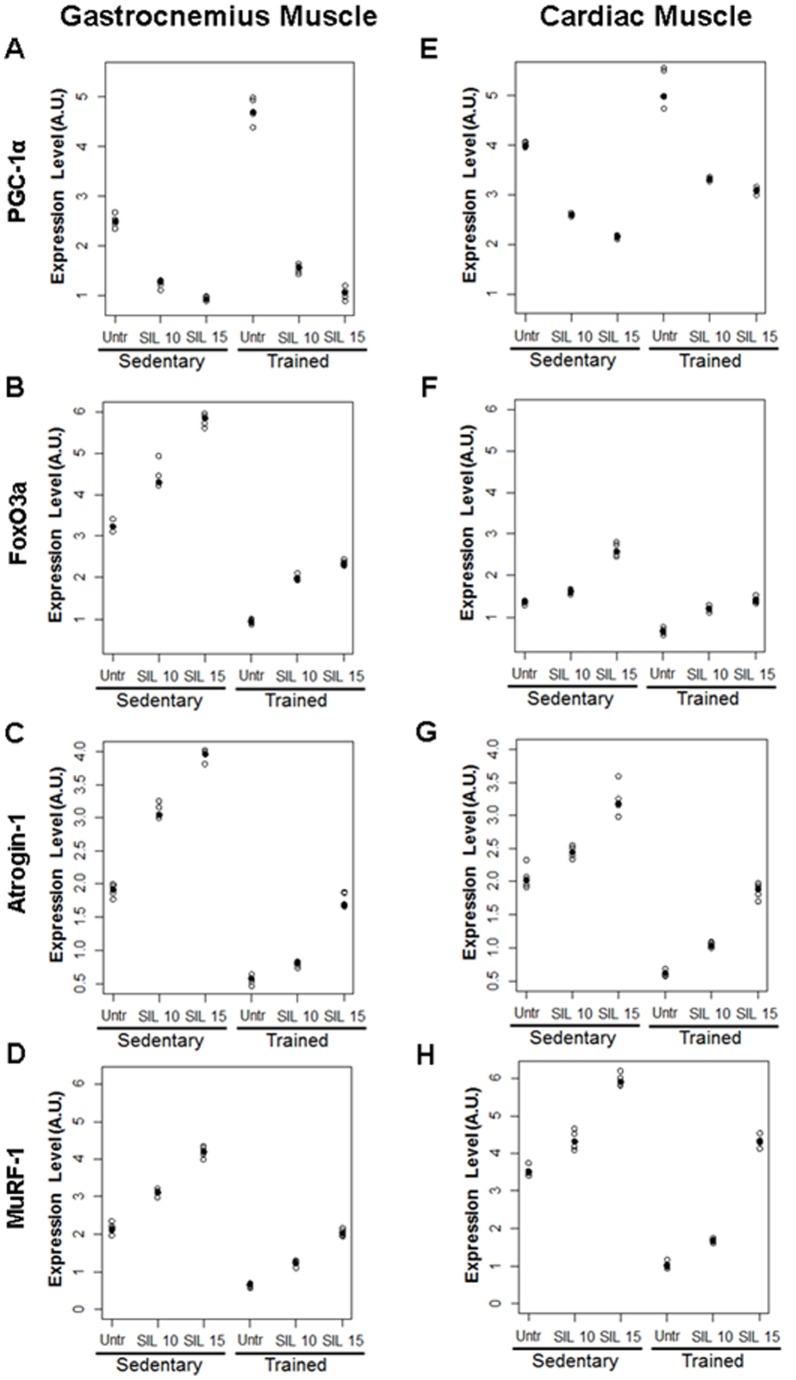
Representative western blot analysis of gastrocnemius and cardiac muscle tissues in sedentary and trained rats. Expression analysis of PGC-1α (A, E), FoxO3a (B, F), Atrogin-1 (C, G) and MuRF-1 (D, H). Values were normalized to GAPDH and were expressed in arbitrary units (A. U.). Individual observed values are plotted by study groups. Median values are highlighted by solid circles.

The effects of exercise training and sildenafil treatment on PGC-1α, FoxO3a, Atrogin-1 and MuRF-1 expressions were confirmed by western blot analysis (Table 4, Figure 4). In fact, sildenafil treatment counteracted in a dose-dependent manner the beneficial effects of training on atrophy in both tissues reducing the expression of PGC-1α (Figure 4A, E), and increasing the expression of the “atrogenes” FoxO3a (Figure 4B, F), Atrogin-1 (Figure 4C, G) and MuRF-1 (Figure 4D, H).

**Table 4:** Representative western blot analysis of gastrocnemius and cardiac muscle tissues in sedentary and trained rats.

	Sedentary Untreated (n = 5)	Sedentary SIL10 (n = 5)	Sedentary SIL15 (n = 5)	Trained Untreated (n = 5)	Trained SIL10 (n = 5)	Trained SIL15 (n = 5)
**Gastrocnemius muscle**						
aPGC-1α	2.50 (2.45–2.53)	1.28 (1.22–1.30)^§^	0.93 (0.90–0.97)^§°^	4.70 (4.65–4.93)^b^	1.56 (1.50–1.59)^b§^	1.06 (0.98–1.10)^b§^
aFoxO3a	3.24 (3.12–3.25)	4.30 (4.22–4.46)*	5.83 (5.71–5.89)^§°^	0.95 (0.93–0.98)^b^	1.98 (1.97–2.01)^b§^	2.33 (2.30–2.37)^b§°^
aAtrogin-1	1.93 (1.87–1.98)	3.05 (3.03–3.15)*	3.95 (3.95–3.98)^§°^	0.58 (0.55–0.65)^b^	0.81 (0.79–0.83)^b^*	1.69 (1.68–1.87)^b§^_°_
aMuRF-1	2.13 (2.11–2.20)	3.12 (3.10–3.15)^§^	4.20 (4.12–4.30)^§°^	0.65 (0.60–0.67)^b^	1.23 (1.10–1.27)^b§^	2.03 (1.98–2.08)^b§°^
**Cardiac muscle**						
aPGC-1α	4.01 (3.98–4.05)	2.60 (2.59–2.64)^§^	2.17 (2.15–2.18)^§°^	5.00 (4.98–5.51)^b^	3.32 (3.32–3.32)^b§^	3.10 (3.08–3.12)^b§^
aFoxO3a	1.36 (1.35–1.37)	1.61 (1.60–1.67)*	2.59 (2.50–2.75)^§°^	0.67 (0.65–0.71)^b^	1.20 (1.19–1.21)^b§^	1.40 (1.39–1.44)^b§†^
Atrogin-1	2.03 (1.97–2.07)	2.45 (2.40–2.50)	3.18 (3.15–3.25)^§°^	0.63 (0.60–0.64)^b^	1.04 (1.03–0.09)^b^	1.90 (1.82–1.94)^b§°^
aMuRF-1	3.50 (3.47–3.54)	4.32 (4.18–4.52)^§^	5.89 (5.82–6.00)^§°^	1.02 (0.99–1.07)^b^	1.68 (1.67–1.71)^b§^	4.32 (4.29–4.35)^b§°^

Data are reported as median and interquartile range.

a Interaction between training and sildenafil dose was statistically significant (sildenafil dose effect must be read separately for sedentary and trained rats).

Training effect: ^b^p<0.001 Trained vs. Sedentary.

Sildenafil dose effect: ^§^p<0.001 vs. Untreated, *p<0.05 vs. Untreated, ^°^p<0.001 vs. SIL10, ^†^p<0.05 vs. SIL10.

**Figure 5:**
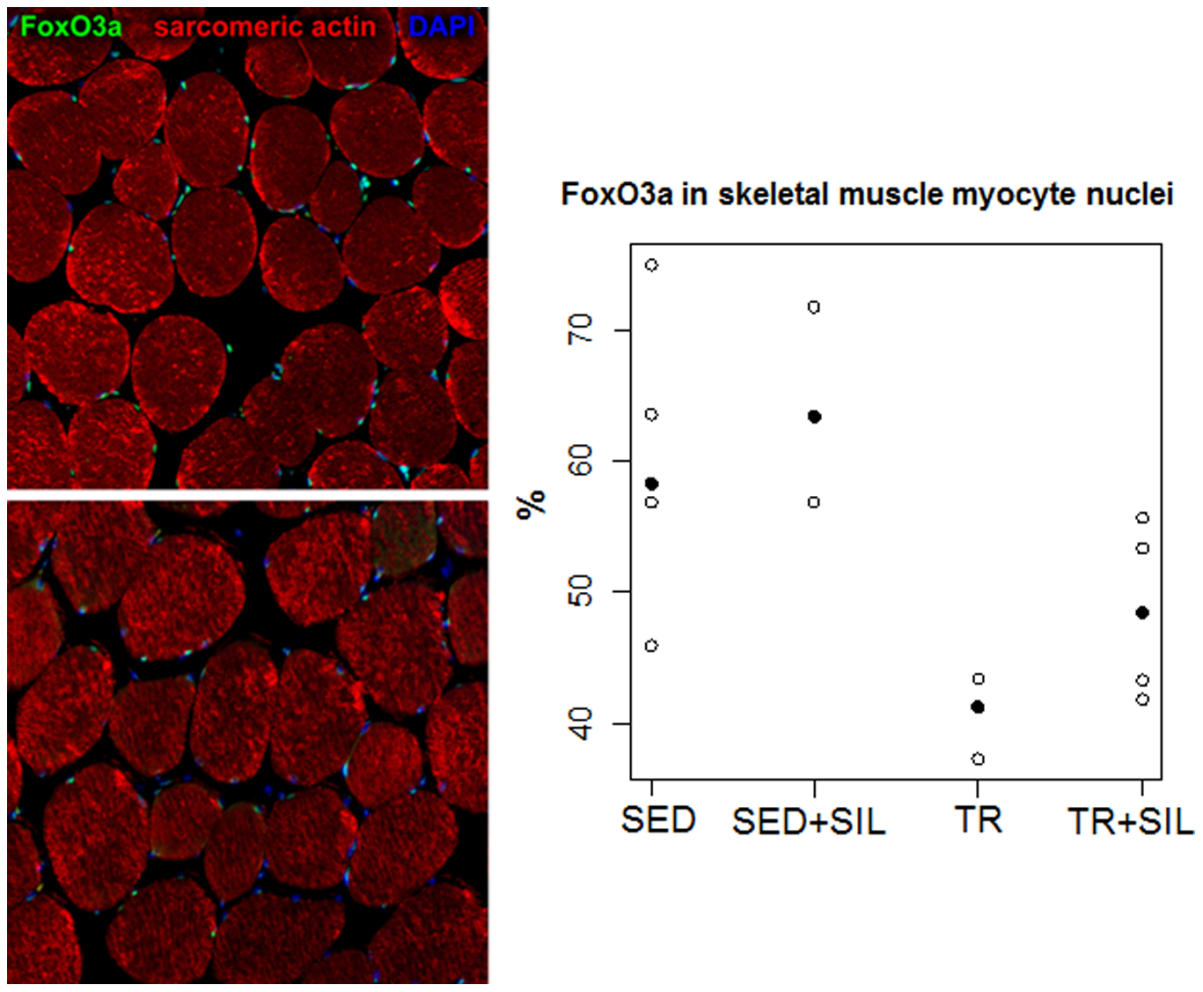
The expression of FoxO3a in the myocyte nuclei of sedentary (upper panel) and trained (lower panel) rats. Myocyte cytoplasm was identified with α-sarcomeric actin antibody labelling (red). Nuclei are stained with 4′,6-diamidino-2-phenylindole (DAPI, blue). Observed percentages of FoxO3a-positive nuclei (green) in untreated and sildenafil-treated sedentary animals (SED and SED+SIL, respectively) and untreated and sildenafil-treated trained rats (TR and TR+SIL) are plotted. Median values are highlighted by solid circles.

**Figure 6:**
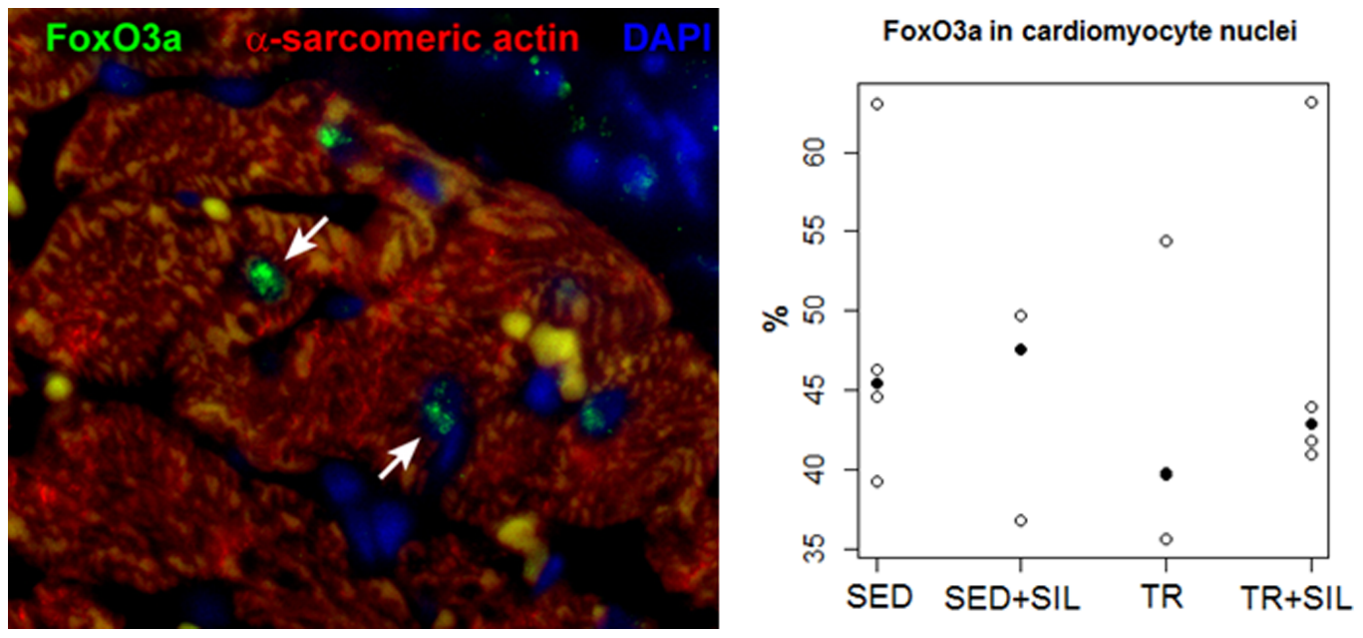
The expression of FoxO3a in the nuclei of left ventricular cardiomyocytes identified with α-sarcomeric actin antibody labeling (red). Nuclei are stained with DAPI (blue). Observed percentages of cardiomyocytes positive for FoxO3a (green, arrows) in the myocardium of untreated and sildenafil-treated sedentary animals (SED and SED+SIL, respectively) and untreated and sildenafil-treated trained rats (TR and TR+SIL) are plotted. Median values are highlighted by solid circles.

The level of FoxO3a activation confirmed by the nuclear localization of this transcription factor in histological sections of gastrocnemius (Figure 5) and heart muscle (Figure 6) of untreated and sildenafil-treated sedentary and trained rats. The expression of FoxO3a in the nuclei of gastrocnemius muscle myocytes was significantly lower in trained rats than in sedentary rats (p<0.01), but there was no effect of sildenafil in both groups (p = 0.27) (Figure 5). However, in the myocardium, neither exercise training nor sildenafil modified the fraction of FoxO3a-positive cardiomyocytes (p = 0.17 and p = 0.45, respectively) (Figure 6).

### Effects of Sildenafil on Gastrocnemius and Cardiac Muscular Hypoxia-responsive Genes in a Rat Model of Prolonged Moderate Exercise

**Figure 7:**
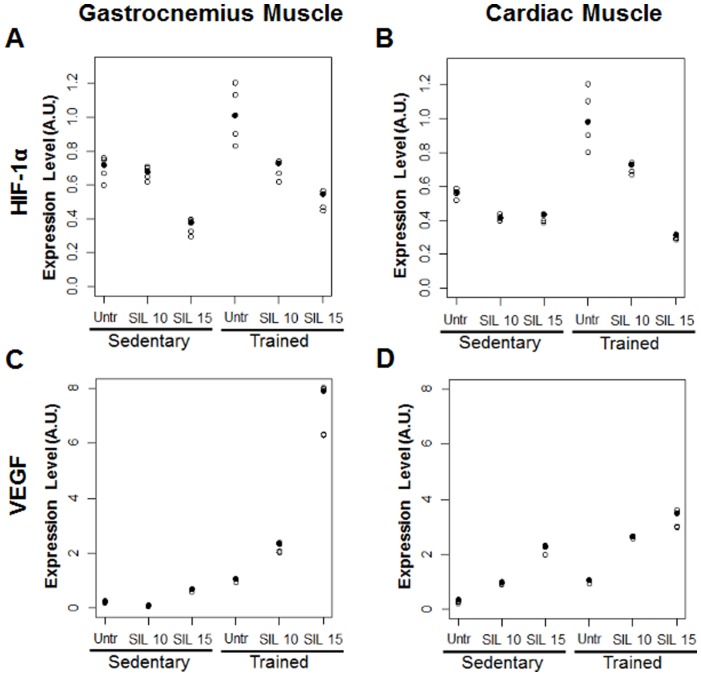
Effects of sildenafil on skeletal and cardiac muscle HIF-1α and VEGF mRNA expression. Top panel (A, B) HIF-1α gene expression; bottom panel (C, D) VEGF gene expression in gastrocnemius and cardiac tissues. Individual observed values are plotted by study groups. Median values are highlighted by solid circles.

The effects of training and sildenafil doses on gastrocnemius and cardiac muscular hypoxia-responsive genes, measured by real-time PCR, are reported in Table 5 and Figure 7. Hypoxia induced by exercise training stimulates activation of the HIF-1α transcription factor that, in turn, induces the expression of several genes such as VEGF. Both HIF-1α (Figure 7A, B) and VEGF (Figure 7C, D) mRNA expression were significantly higher in skeletal and cardiac tissues of trained rats compared with sedentary rats (p<0.001). These data suggest that the exercise-induced metabolic stress, including local hypoxia, is a primary stimulus for the induction of increased capillarization and oxygen supply.

**Table 5:** Effects of sildenafil on gastrocnemius and cardiac muscle hypoxia-responsive genes.

	Sedentary Untreated (n = 5)	Sedentary SIL10 (n = 5)	Sedentary SIL15 (n = 5)	Trained Untreated (n = 5)	Trained SIL10 (n = 5)	Trained SIL15 (n = 5)
**Gastrocnemius muscle**						
aHIF-1α	0.72 (0.67–0.75)	0.68 (0.65–0.70)	0.38 (0.33–0.39)^§°^	1.01 (0.90–1.13)^b^	0.73 (0.67–0.74)^b^*	0.55 (0.47–0.56)^b§^
aVEGF	0.25 (0.23–0.26)	0.10 (0.09–0.10)^§^	0.68 (0.61–0.68)^§°^	1.05 (0.95–1.06)^b^	2.34 (2.07–2.38)^b§^	7.89 (6.32–7.96)^b§°^
**Cardiac muscle**						
aHIF-1α	0.57 (0.56–0.59)	0.42 (0.41–0.42)^§^	0.43 (0.40–0.44)^§^	0.98 (0.90–1.10)^b^	0.73 (0.69–0.73)^b§^	0.31 (0.30–0.32)^b§°^
aVEGF	0.35 (0.30–0.35)	0.98 (0.90–0.98)^§^	2.27 (2.26–2.29)^§°^	1.05 (0.95–1.07)^b^	2.65 (2.64–2.68)^b§^	3.51 (3.03–3.59)^b§°^

Data are reported as median and interquartile range.

a Interaction between training and sildenafil dose was statistically significant (sildenafil dose effect must be read separately for sedentary and trained rats).

Training effect: ^b^p<0.001 Trained vs. Sedentary.

Sildenafil dose effect: ^§^p<0.001 vs. Untreated, *p<0.05 vs. Untreated, ^°^p<0.001 vs. SIL10.

On the other hand, sildenafil significantly and dose-dependently increased the mRNA levels of the angiogenetic factor VEGF in both tissues while counteracted in a dose-dependent manner the effects of training on HIF-1α mRNA expression in both gastrocnemius and heart muscle, mainly in trained rats.

### Effects of Sildenafil on Vascular Growth in an Experimental Model of Prolonged Moderate Exercise

**Figure 8:**
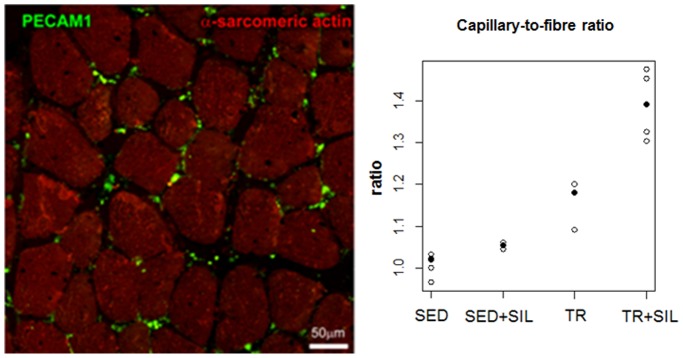
Example of capillaries labelled with PECAM1 antibody (green) in a skeletal muscle tissue (α-sarcomeric actin antibody, red). Observed capillary-to-fibre ratio in untreated and sildenafil-treated sedentary animals (SED and SED+SIL, respectively) and untreated and sildenafil-treated trained rats (TR and TR+SIL) are plotted. Median values are highlighted by solid circles.

To evaluate the effects of training and to determine whether sildenafil affects vascular growth the capillary-to-fibre ratio was calculated (Figure 8). Exercise resulted in a significant increase in the capillary-to-fibre ratio in skeletal muscle (p<0.001) and treatment with sildenafil further significantly increased this parameter only in trained group (p<0.05).

### Effects of Sildenafil on Myocardial Capillary Density after 8 Weeks of Exercise Training

**Figure 9:**
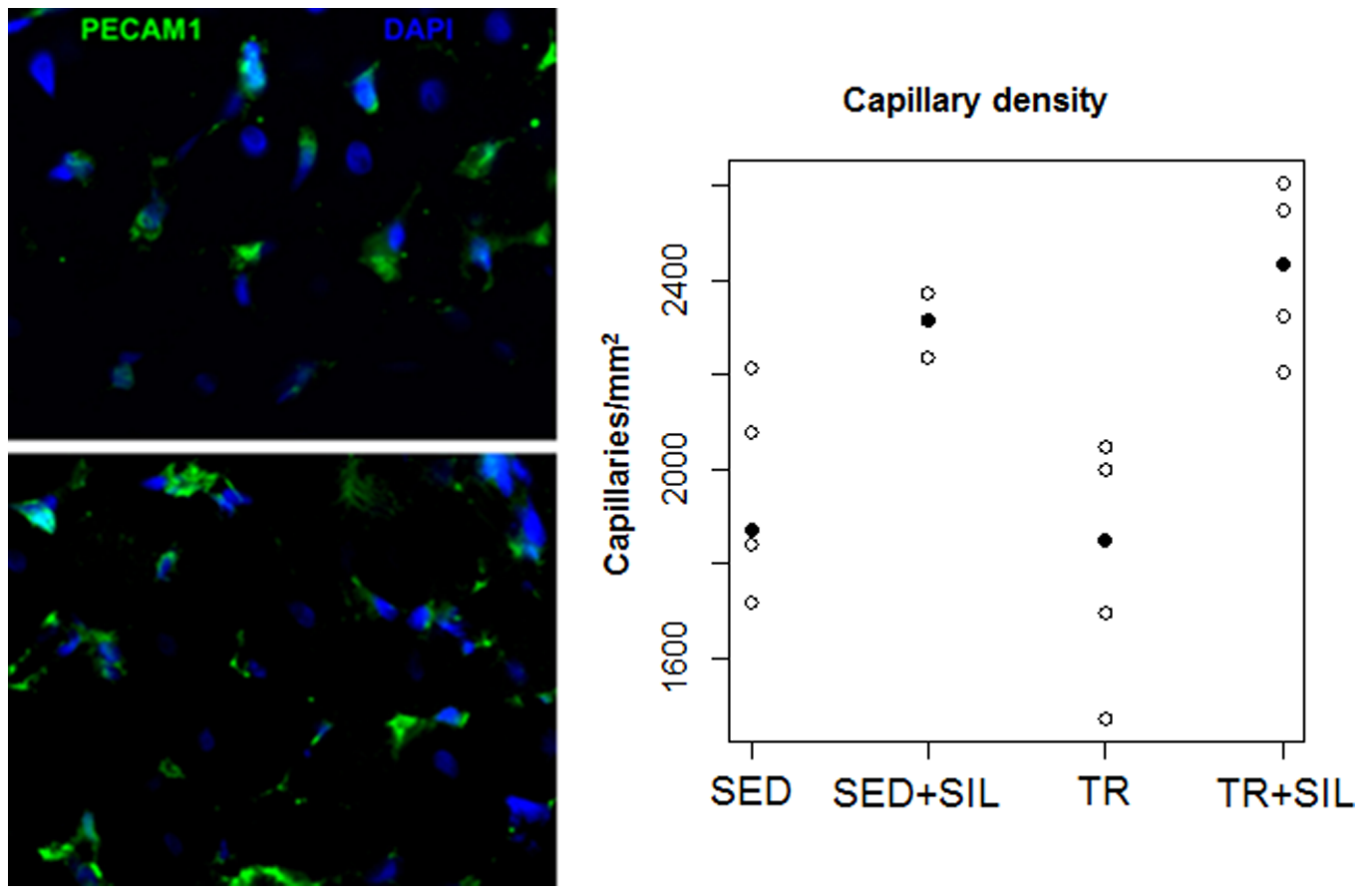
Examples of coronary capillaries labelled with PECAM1 antibody (green) in the myocardium of rats. Sedentary animal (upper panel) and trained rat treated with sildenafil (lower panel). Nuclei are stained with DAPI (blue). Observed quantification of the number of coronary capillaries in untreated and sildenafil-treated sedentary animals (SED and SED+SIL, respectively) and untreated and sildenafil-treated trained rats (TR and TR+SIL) are plotted. Median values are highlighted by solid circles.

To determine whether sildenafil alone or in combination with exercise training affected coronary vasculature, myocardial capillary density was analysed (Figure 9). Exercise training did not affect coronary microvasculature. However, an increase in capillary density was detected in the myocardium of animals that received sildenafil (p<0.001), in the same manner in trained and sedentary group (interaction p = 0.45).

### Effects of Sildenafil on the Modulation of Muscle Fibre Size after Exercise Training

**Figure 10:**
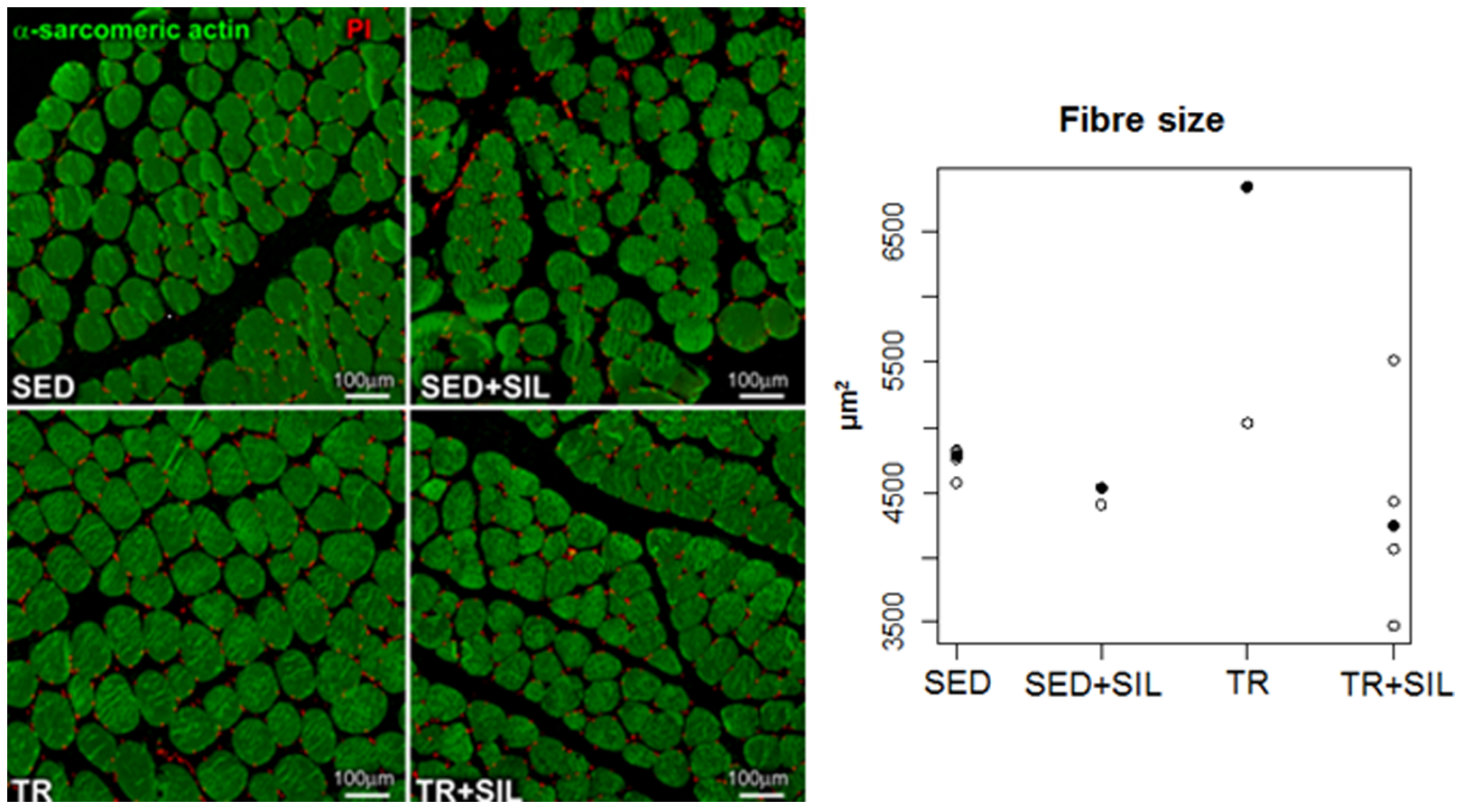
Cross-section of muscle fibre of a gastrocnemius muscle. Myocyte cytoplasm was identified with α-sarcomeric actin antibody staining (green). Cell nuclei are labelled with propidium iodide (PI; red). Observed quantification of fibre cross-sectional area in untreated and sildenafil-treated sedentary animals (SED and SED+SIL, respectively) and untreated and sildenafil-treated trained rats (TR and TR+SIL) are plotted. Median values are highlighted by solid circles.

Next muscle fibre size was examined in all experimental groups to determine the net effects of the modulation of anabolic and catabolic pathways in the skeletal muscle tissue induced by training and sildenafil. As shown in Figure 10, exercise training resulted in an increase in fibre size measured in a cross-sectional area of skeletal muscle tissue (p<0.05). However, when trained animals were repeatedly exposed to sildenafil, the hypertrophic response of myocytes was not present. This observation indicates that the pro-hypertrophy signalling activated by the physical activity was suppressed by the atrophy-related pathways stimulated by the drug.

## Discussion

In recent years, many attempts have been made to identify new therapeutic applications of sildenafil [15], [17], which is currently used to treat erectile dysfunction and pulmonary hypertension. Moreover, sildenafil is increasingly being used to enhance sports performance, although it is not included in the World Anti-Doping Agency’s list of prohibited substances. The aim of the present study was to evaluate the effects of moderate exercise training and of a repeat administration of two doses of sildenafil on the gastrocnemius and cardiac muscles of rats. In particular, we investigated the role of sildenafil in the adaptive response of muscle to exercise by evaluating the effects of this drug on atrophic and angiogenetic events. Our data demonstrate that sildenafil: 1) decreased the expression of the PGC-1α gene, which protects against atrophy, and increased the expression of the atrophy-related genes FoxO3a, Atrogin-1 and MuRF-1 thereby reducing the skeletal muscle hypertrophy induced by exercise; 2) counteracted the effects of training on the expression of HIF-1α gene, which is involved in hypoxia, and 3) enhanced the proangiogenetic effect of exercise thereby increasing VEGF expression and capillary density.

Exercise training is believed to ameliorate quality of life by improving the blood lipid profile, by reducing sympathetic activity and by increasing the defence system [36], [37]. Our model of exercise training, in line with our previous data and those of another group obtained with the treadmill [28], [38], did not induce significant changes in cardiac physiology or morphology. However, a repeat administration of sildenafil induced hypotension and increased the EF in sedentary and trained rats.

We found that sildenafil induced the atrophy pathways. It is well recognised that exercise strongly induces PGC-1α [39]. Moreover, PGC-1α counteracts the FoxO3a-dependent transcription of Atrogin-1 and MuRF-1, two genes up-regulated in different models of muscular atrophy [40]. We confirm the induction of PGC-1α expression after training and we found that a repeat administration of sildenafil promoted muscular atrophy both by down-regulating the anti-atrophy gene PGC-1α and by up-regulating pro-atrophy genes, thereby counteracting the increase in fibre size induced by our exercise training model. Decreased expression of PGC-1α is linked to a reduction of mitochondrial biogenesis, oxidative phosphorylation and respiration [41]. The dysregulation of the expression of genes involved in muscle homeostasis could be related to the reduced exercise capacity we observed in sildenafil-treated rats at the end of treatment. Importantly, the effects of sildenafil on PGC-1α expression were more evident in trained rats, which suggests that exercise and pharmacological treatment exerted a synergistic effect. Exercise training affected not only muscle atrophy but also the angiogenic process, which is regulated by such mediators as VEGF and HIF-1α that are released in the tissues surrounding the small vessels. VEGF plays a key role in angiogenic processes in skeletal muscle and myocardium [42]. One of the mechanisms involved in rapid activation of VEGF expression is a fall in intracellular oxygen level during exercise, which results in enhanced expression of HIF-1α [30], [43]. As expected, also our experimental model of training induced a significant increase in HIF-1α expression in skeletal and cardiac muscles. In trained rats, sildenafil restored HIF-1α expression. These observations are in line with the positive effects that sildenafil exerts on tissue oxygenation in such diseases as pulmonary artery hypertension and myocardial ischemia [15], [17].

Another notable finding of our study is the synergic effect of exercise training and sildenafil on angiogenesis. In fact, exercise induced VEGF expression, and the concomitant administration of sildenafil significantly and dose-dependently enhanced this effect. Previous studies have described the proangiogenetic effect of sildenafil *in vitro*, in cultured endothelial cells, and *in vivo*, at both capillary and arteriolar levels in an experimental model of ischemia reperfusion [44], [45]. The latter studies coincide with our finding that sildenafil exerts a positive effect on the number of coronary capillaries.

### Conclusions

A repeat administration of sildenafil exerted beneficial and negative effects on skeletal and cardiac muscle of trained rats. Interestingly, treatment with sildenafil for one week dose-dependently enhanced muscle atrophy in both sedentary and trained rats. Therefore, this compound seems to counteract the beneficial effects of exercise on muscle strengthening. On the other hand, sildenafil potentiated the effects of exercise on angiogenesis and hypoxia. It would be interesting to evaluate the effects of high doses of sildenafil in models of exercise training devised to determine its effect as a performance-enhancing substance in athletes.
